# Bilateral Vocal Cord Fibrosis: A Delayed Complication of Button Battery Ingestion

**DOI:** 10.7759/cureus.25721

**Published:** 2022-06-07

**Authors:** Norbert F Banhidy, Shiraz Jamshaid, Reshma Ghedia, Nara Orban

**Affiliations:** 1 Otolaryngology - Head and Neck Surgery, Royal London Hospital, London, GBR; 2 Otolaryngology, Royal London Hospital, London, GBR

**Keywords:** paediatric surgery, paediatric otorhinolaryngology, electromyography (emg), caustic ingestion injury, bilateral vocal cord paralysis

## Abstract

A 14-month-old girl initially presented to the Accident and Emergency (A&E) department following a choking episode and subsequent vomiting. The child left the department before being seen but re-presented the following morning with stridor, drooling, and increased work of breathing. A chest and lateral neck soft tissue X-ray performed in the A&E department revealed an ingested button battery in the oesophagus. Emergency oesophagoscopy was performed and a 22 mm button battery was removed from the oesophagus at the level of the cricopharyngeus muscle, with no immediate complications. Following extubation, the patient was initially well but later required a prolonged hospital stay due to recurrent episodes of stridor, voice changes and aspiration pneumonia. Follow-up microlaryngoscopy and laryngeal electromyography (EMG) diagnosed bilateral vocal cord palsy and cricoarytenoid fibrosis. This case highlights the need for increased public awareness, urgent diagnosis and standardised management of battery ingestion, and discusses the potential for the development of serious latent complications.

## Introduction

The ingestion of button batteries found in everyday consumer electronic devices accounts for over 3300 hospital presentations each year in the USA [1]. The development of physically larger and more powerful batteries in recent years has led to an increasing frequency of morbidity and mortality associated with battery ingestion, especially in children under the age of six [2]. Seventy per cent (70%) of ingested button battery lodgement occurs in the upper oesophagus, leading to complications, including but not limited to oesophageal perforation and strictures, recurrent laryngeal nerve injury, tracheo-oesophagal fistulas, and aorto-oesophagal fistulas [3]. The mechanism of injury is a result of direct electrical current to structures adjacent to the battery [4]. Upon contact with an acidic environment, such as the gastrointestinal tract, the negative battery pole, identifiable as the narrowest side of the battery, generates an electrical current, which leads to electrolysis, hydroxide production, and ultimately necrosis of surrounding tissue [5]. Urgent recognition and removal of the battery are paramount, as the severity of the injury is proportional to the time spent in contact with the tissue, with localised necrosis occurring in as little as two hours [6-7]. Diagnosis is often delayed and complications can arise insidiously long after the removal of the battery, as in this case of a child who developed bilateral vocal cord fibrosis following delayed presentation of an ingested button battery.

## Case presentation

Presentation

A 14-month-old girl initially presented to the accident and emergency (A&E) department following a choking episode and subsequent vomiting. The child left the department before being seen by a doctor due to the long waiting time and spontaneous improvement of her symptoms. The following morning, the child returned to the A&E distressed with stridor, drooling and increased work of breathing. A series of X-rays were taken upon suspicion of an ingested foreign body.

The patient was born at term following an uncomplicated vaginal delivery. She was previously fit and well with no significant medical history, awaiting her one-year immunisations. She lived with both parents.

Investigations

**Figure 1:**
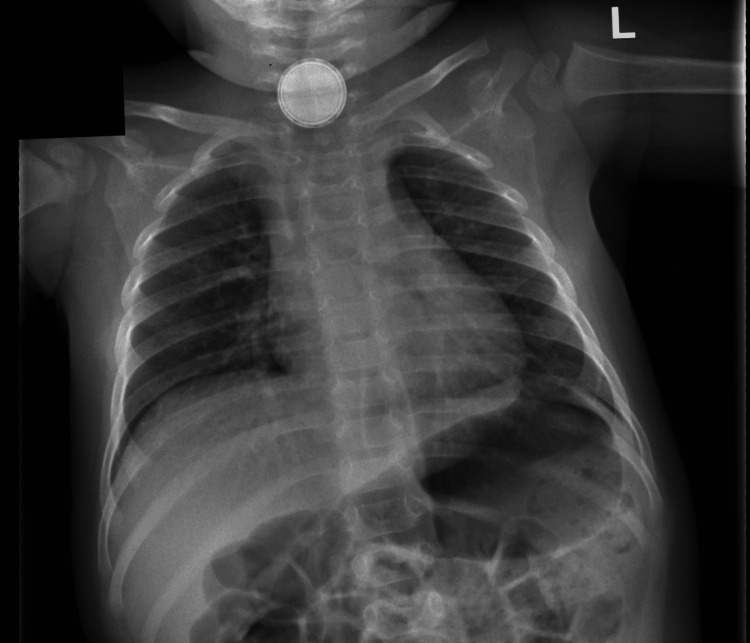
Anteroposterior (AP) erect chest X-ray

**Figure 2:**
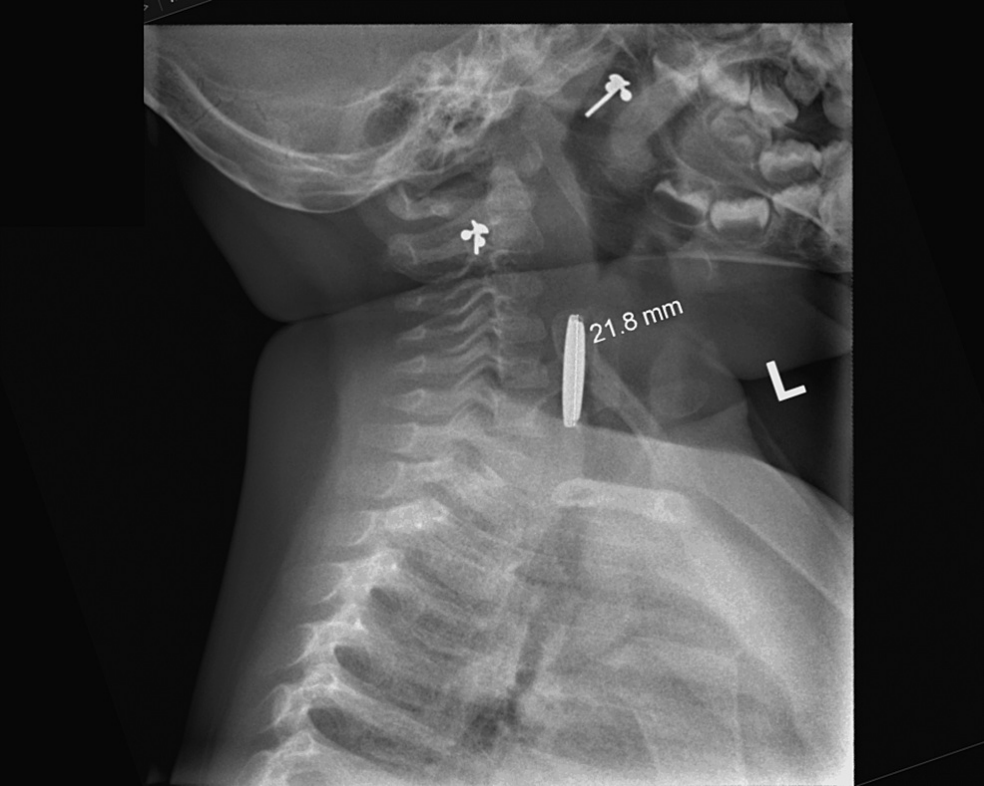
Lateral soft tissue neck X-ray

A chest and lateral neck soft tissue X-ray performed in the A&E department demonstrated a round radio-opaque foreign body overlying the anterior aspect of the lower cervical spine (Figure 1 and Figure 2). The object measured 22 mm in diameter and demonstrated the step-off sign on lateral inspection and the halo sign on anterior inspection. These findings helped confirm the identity of the ingested foreign body as a button battery.

Management

**Figure 3:**
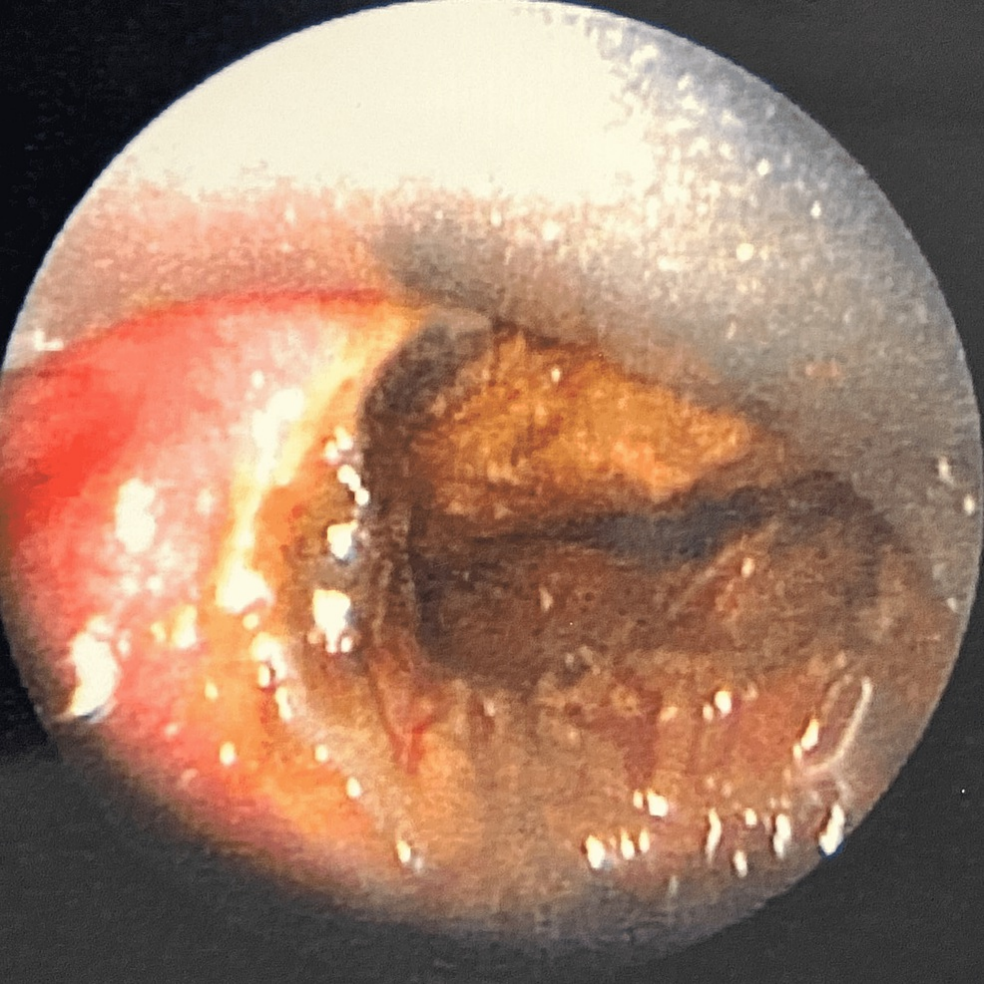
Endoscopic view of the button battery and surrounding oesophageal damage

**Figure 4:**
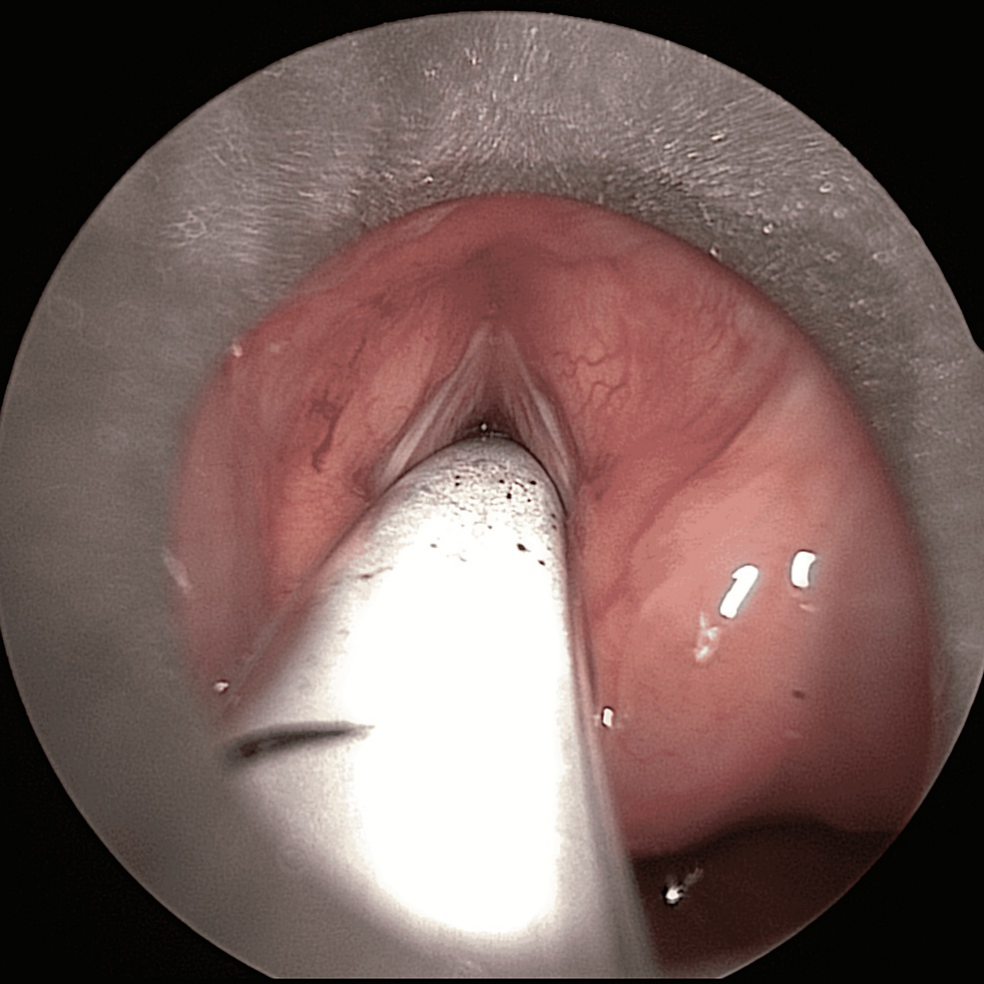
Endoscopic view of the glottis

The child was commenced on intravenous (IV) dexamethasone, co-amoxiclav, and esomeprazole in the emergency department, and following confirmation of the button battery ingestion on X-ray, an emergency oesophagoscopy was performed by the ear, nose and throat (ENT) surgeon. A 22 mm button battery was removed from the oesophagus at the level of the cricopharyngeus muscle. Circumferential oesophageal charring was noted at the level of the battery, with no significant glottic or supraglottic oedema at the time of inspection (Figure 3 and Figure 4). In light of the extensive oesophageal burns, the decision was made to keep the child intubated due to the risk of delayed laryngeal oedema. The patient was transferred to a tertiary centre for subsequent relook oesophagogastroscopy, microlaryngoscopy and bronchoscopy under the joint care of the paediatric surgery and ENT teams.

Microlaryngoscopy and bronchoscopy the following day revealed moderate epiglottic inflammation, normal appearance of the vocal cords, no subglottic stenosis and only minimal erythema and oedema on the posterior wall of the subglottis. The trachea and bronchi appeared normal. Oesophagogastroscopy revealed small amounts of black debris in the stomach and distal oesophagus, along with a 1 cm length of black discolouration extending ⅔rds around the oesophagus, 10 cm from the teeth. There appeared to be no reduction in the diameter of the oesophageal lumen.

Outcome and follow-up

The child was successfully extubated two days after initial intubation. Minimal voice hoarseness was noted but no other immediate complications arose. Nasogastric feeding commenced upon extubation along with dietitian and speech and language input. Steroids and proton pump inhibitors were weaned the following day.

The patient redeveloped stridor eight days following extubation. She was recommenced on IV dexamethasone and prescribed nebulised adrenaline as needed. Bedside flexible nasal endoscopy at the time revealed normal vocal cord appearance and movement with no significant supraglottic or glottic oedema. A chest X-ray was performed, which demonstrated right lower zone consolidation in keeping with aspiration pneumonia, and the patient was subsequently commenced on IV co-amoxiclav. The patient clinically improved and was discharged home a few days later with a course of oral antibiotics and a view to returning for an elective microlaryngoscopy and bronchoscopy.

The patient had brief readmission to the hospital following initial discharge due to noisy breathing. She remained clinically well with normal observations and maintained adequate oxygen saturation on room air. A flexible nasendoscopy revealed only mild arytenoid swelling but normal laryngeal airway patency, vocal cord position and movements. Following review by both the ENT and paediatric teams, the patient was discharged with a short course of oral dexamethasone.

An elective microlaryngoscopy and bronchoscopy were performed one month following the initial presentation, which revealed a small degree of paradoxical movement of the vocal cords. A subsequent magnetic resonance imaging (MRI) scan of the neck demonstrated no brainstem abnormality and the courses of the vagus nerves and recurrent laryngeal nerves in the neck and in the superior mediastinum appeared satisfactory. 

Two weeks later, the patient re-presented with stridor, increased work of breathing and desaturations, ultimately requiring intubation. The following day, a microlaryngoscopy and tracheoscopy demonstrated improved, but still reduced vocal cord movement. The patient was extubated but continued to have stridor at rest and the decision was made to commence her on continuous positive airway pressure (CPAP) ventilation to support her breathing. The patient was subsequently discharged with home CPAP.

One month following discharge, the patient underwent an elective diagnostic microlaryngoscopy and laryngeal electromyography (L-EMG). The vocal cords demonstrated no movement bilaterally and bilateral posterior cricoarytenoid joint fixation was noted on microlaryngoscopy, indicative of bilateral vocal cord fibrosis. Intraoperative L-EMG demonstrated low amplitude potentials of the vocal folds bilaterally indicating bilateral denervation and palsy of the vocal cords.

Since the diagnosis of bilateral vocal cord fibrosis, the patient has had to undergo multiple procedures, including an initially unsuccessful tracheo-laryngeal reconstruction and subsequent pericardial patch repair to her trachea. Due to ongoing high aspiration risk and poor feeding, the patient underwent a percutaneous endoscopic gastrostomy (PEG), and she continues to be fed via the PEG tube. She also requires ongoing respiratory support due to frequent deteriorations in her breathing.

## Discussion

This case highlights the devastating consequences of the late recognition of button battery ingestion. The majority of patients presenting to the A&E following battery ingestion are asymptomatic, with non-specific symptoms such as nausea, vomiting, dysphagia or irritability only occurring in 10-20% [5]. This is further compounded by a lack of public awareness of the potential severity of the injuries caused by batteries, leading to delayed presentations [3]. Symptoms of serious complications only present hours to days following initial ingestion and may include haematemesis, chest or abdominal pain, stridor or hoarseness [8]. Consequently, triage and treatment algorithms indicate the need for immediate serial X-rays (including neck, chest and abdomen) in any suspected or known cases of battery ingestion to aid in swift diagnosis [4].

Diagnosis must be followed by immediate endoscopic removal of the battery by an ENT surgeon to allow for direct visualisation of the battery and associated tissue injury [4]. Decreasing the time to endoscopic intervention is crucial, and this may be facilitated by activating a local trauma protocol to ensure expedient evaluation and shortened time to removal [9]. Consideration should also be given to opening up a second emergency theatre if required to expedite battery removal.

Major complications from button battery ingestion may not be immediately noticeable upon initial endoscopic evaluation and removal, therefore steps must be taken early to anticipate and prevent harmful sequelae. Specific complications should be anticipated based on battery position, orientation, and the time taken for removal. A second relook oesophagoscopy is recommended within two to four days of battery removal to aid prognostication [10]. Serial cross-sectional imaging, such as computerized tomography (CT) or MRI scans, may also be useful to identify the progression or resolution of tissue injury [11]. Early multidisciplinary team (MDT) input must also be sought to adequately manage all potential complications. This often includes speech and language therapists, paediatrics, dietitians, ENT, and paediatric surgeons [12].

Vocal cord palsy and associated cricoarytenoid fibrosis have been linked to button battery ingestion, especially when the battery has been found to be in close proximity to the larynx [8,13]. Voice changes, symptoms of aspiration or stridor following extubation may all indicate the presence of vocal cord damage. Flexible nasendoscopy by the bedside should be performed to assess vocal cord position and movement. Microlaryngoscopy allows for the manipulation of the arytenoid cartilage to assess for vocal cord fibrosis. Laryngeal EMG may also be considered to aid in the diagnosis and prognostication of vocal cord palsy [4].

## Conclusions

Button battery ingestion remains a major public health issue in the United Kingdom and vocal cord palsy secondary to cricoarytenoid fibrosis is a serious long-term complication of button battery chemical necrosis. Accurate data on the incidence of button battery ingestion and associated morbidity/mortality is still lacking. Presentations suggestive of button battery ingestions require urgent X-ray imaging and review by a senior doctor. Although local hospital guidelines are available in aiding the A&E management of such cases, a standardised national clinical decision support tool is still lacking for cases of suspected or known battery ingestion. Finally, the severity of harm caused by ingested button batteries remains poorly understood by the public, and public education about the consequences of button battery ingestion needs to be improved.
